# LIM-domain proteins TRIP6 and LPP associate with shelterin to mediate telomere protection

**DOI:** 10.18632/aging.100170

**Published:** 2010-07-14

**Authors:** Samantha A. Sheppard, Diego Loayza

**Affiliations:** Department of Biological Sciences Hunter College 695 Park Avenue New York, NY 10065

**Keywords:** Telomere, shelterin, POT1, LIM domain, TRIP6, LPP, p53BP1

## Abstract

POT1 is the single stranded telomeric overhang binding protein, and is part of the shelterin complex, a group of six proteins essential for proper telomere function. The reduction or abrogation of POT1 DNA binding activity in mammalian cells results in telomere elongation, or activation of the ATR DNA damage response at telomeres. Therefore, overhang binding represents the functionally relevant activity of POT1. To better understand the roles of POT1, we sought to isolate proteins that interact with the DNA binding domain of the protein. A yeast two-hybrid screen was implemented using a C-terminal truncation termed POT1ΔC, retaining the DNA binding domain. This screen yielded a partial cDNA corresponding to TRIP6, a member of the LIM domain protein family. TRIP6 could co-immunoprecipitate with POT1, TRF2 and TIN2 in human cells, arguing for association with the whole shelterin complex, and was detected at telomeres by ChIP. TRIP6 depletion by siRNA led to the induction of telomere dysfunction induced foci (TIFs), indicating a role in telomere protection. A closely related LIM domain protein, LPP, was also found at telomeres and was also important for repressing the DNA damage response. We propose that TRIP6 and LPP are both required for telomere protection.

## Introduction

Telomeres are essential chromosomal elements, which ensure proper replication and protection of chromosome ends. Human telomeres are constituted by 2-12 kb of double-stranded TTAGGG repeats and present a single-stranded overhang of about 150 nucleotides. Telomeres prevent an inappropriate DNA damage response by recruiting a six-protein complex called shelterin, which is able to inhibit the induction of ATM and ATR responses [1]. They are also part of a feedback loop that ultimately regulates the ability of telomerase to add TTAGGG repeats preferentially to short telomeres [2]. Both of these activities require binding of POT1, one of the shelterin components, to the telomeric overhang [3].

The assembly of shelterin on the telomere is initiated by TRF1 and TRF2, which bind double stranded TTAGGG repeats directly through a MYB-type DNA binding domain. They in turn recruit RAP1, TIN2, TPP1 and POT1 [1]. TIN2 has the ability to interact with both TRF1 and TRF2 simultaneously, and additionally recruits TPP1/POT1 to the complex. The TPP1/POT1 heterodimer is believed to have a dual role in the regulation of telomerase. POT1 itself is able to prevent or limit telomere elongation through its DNA binding activity [4], and TPP1 possesses a recruitment domain for telomerase providing a link between the enzyme and the chromosome's end [5]. It is with two N-terminal OB folds that POT1 binds the telomeric overhang, with high affinity and sequence specificity [6,7].

Extensive analysis of the shelterin complex by mass spectrometry and proteomics, performed by a number of laboratories [3,8-10] has led to the discovery of shelterin as a stable complex of six proteins, and in some cases, has identified components transiently associated with telomeres (eg. the MRE11 complex [11]). However, some other components known to impact on telomere function, including telomerase itself, have been difficult to detect at telomeres by biochemical methods. These may not be associated with telomeres throughout the cell cycle, and are recruited through poorly understood regulatory events. For instance, the WRN helicase, mutated in the aging disease Werner syndrome, was shown to associate with telomeres in S-phase and to participate in lagging strand DNA synthesis [12]. Thus, some activities, although only transiently associated with shelterin, may be important to mediate its role in telomere function. The currently established shelterin-associated components are recruited through structurally related domains in TRF1 and TRF2 by recognition of F/Y-X-L-X-P docking sites [13]. Another important interaction at the telomere involves the OB fold of TPP1, proposed to be important for the recruitment of telomerase at chromosome ends [5].

POT1 regulates telomere length through its overhang binding activity and mediates telomere protection through inhibition of the checkpoint kinase ATR [1]. POT1 is composed of two functional domains. The first domain lies in the C terminus, and mediates the recruitment of POT1 to telomeres through an interaction with TPP1 [14,15]. The second domain is the N-terminal DNA binding domain constituted by two OB folds, responsible for binding the telomeric overhang with high affinity and sequence specificity [7]. The two POT1 OB folds span the first 299 amino acids of the protein [7]. It is through the binding of the telomeric overhang that POT1 exerts its biological function at telomeres.

The DNA binding activity of POT1 was shown, in the mouse conditional knock out system, to mediate repression of the ATR kinase [16,17], itself an important component of the DNA damage response. This response, which is detected upon the removal of POT1 in mouse cells, results in the convergence and accumulation of DNA damage proteins at telomeres, which are in this case "sensed" as damaged DNA. For instance, DNA damage proteins p53BP1, γH2AX [18], and MDC1 [19] can be detected as foci co-localizing with telomeres. Ultimately, telomere deprotection can lead to extensive end-to-end telomere fusions, a catastrophic cellular event. The initial convergence of proteins involved in the DNA damage response at telomeres leads to the formation of TIFs, (telomere dysfunction-induced foci).

Another important role for POT1 is the cis-inhibition of telomerase. Depletion of POT1 by siRNA leads to elongation of telomeres in telomerase-positive cells [20], placing POT1 in a cis-inhibiting pathway of inhibition of telomerase as a part of the shelterin complex. The engagement of POT1 with the overhang is essential for this inhibitory role, because expression of a N-terminal truncation of the DNA binding domain leads to extensive telomere elongation [4,21].

In vitro systems have demonstrated a positive role for POT1 and the POT1-TPP1 heterodimer in telomerase activity on a model telomere seed [22]. POT1 by itself can lead to increased telomerase recruitment depending on the distance between the binding site and the 3'end of the DNA. More recently, the POT1-TPP1 dimer has been shown to increase repeat addition processivity of the enzyme through a domain in TPP1 [23]. TPP1 itself has been proposed to directly recruit telomerase to telomeres through an OB fold present in the molecule [5]. Therefore, POT1-TPP1 has a dual role in telomerase regulation: a positive role through recruitment and enzymatic regulation of the enzyme, and a negative role through overhang binding activity. These complex activities of POT1 could be regulated in vivo by yet unknown factors.

Since the DNA binding domain of POT1 is essential for both telomere length control and the inhibition of the DNA damage response at telomeres, protein associations with this domain are of great interest to explore. For instance, the roles of POT1 in inhibiting telomerase and the ATR kinase could be mediated by a factor that could either modulate the overhang binding of POT1, or act as a mediator molecule once POT1 has engaged on the DNA.

In this study, we sought to isolate POT1-associated factors that interact with the DNA binding domain of the molecule, therefore expected to play a role in the function of POT1 and not its recruitment. We employed the yeast two-hybrid system to discover potential novel shelterin-associated proteins. To that end, we targeted the screen for candidates binding to the N-terminal domain of POT1, containing the two DNA binding OB folds. We report on the finding that the LIM domain proteins TRIP6 can interact with the POT1 DNA binding domain by two-hybrid, an interaction that we confirmed in human cells. TRIP6, initially identified as a Thyroid Receptor Interacting Protein, met the criterion of binding specifically the N-terminus of POT1 by yeast two-hybrid analysis, and can be detected in a complex with POT1 and other shelterin components in human cells. TRIP6 was previously implicated in cytoskeletal rearrangements and in transcriptional control [24]. LIM domains are known protein interaction domains that present distinctive loops defined by interactions between Cys and His residues coordinating a Zn ion, and define a family of proteins subdivided in specifics groups (reviewed in [25]). Our data is compatible with TRIP6 being transiently associated with telomeres, an association which is readily detected by chromatin immunoprecipitation. We report that the closely related LIM protein LPP was also found in a complex with shelterin components. We found that single depletion of TRIP6 or LPP leads to TIF formation detected by accumulation of p53BP1 at telomeres. Based on our results, we propose that TRIP6 and LPP are both important for telomere protection.

## Results

### Choice of the POT1 bait and yeast two-hybrid screen

**Figure 1.**
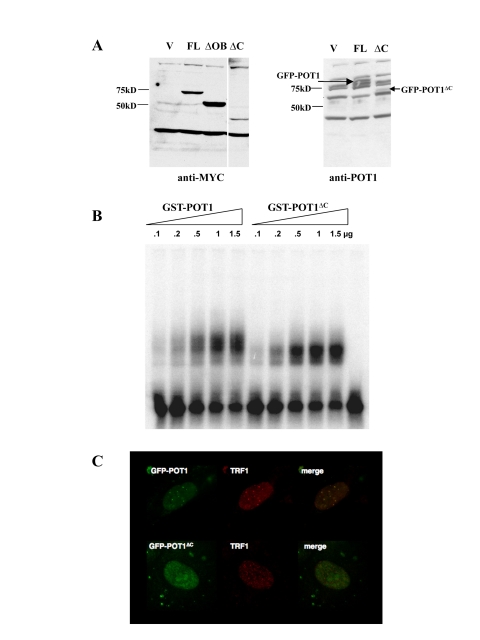
Localization and DNA binding activity of the POT1 ^∆^^C ^allele. (**A**)  Expression levels of MYC-or GFP-tagged alleles in HTC75 cells. The full-length (FL, 71kD), POT1∆OB (∆OB, MW 57kD) and POT1∆C(∆C, MW 43kD) are shown along a vector-only control. Blots probed with the 9E10 (anti-MYC) (left) or 978 (anti-POT1) antibodies (right) are shown. (**B**) Gel shift assay for GST-POT1 and GST-POT1∆C. A ^32^P-labelled oligonucleo-tide containing the POT1 minimal binding site was incubated with the amounts of GST fusion protein shown on top. The free probe is visible at the bottom of the autoradiogram. (**C**) Intranuclear localization of GFP-NLS-POT1 and GFP-NLS-POT1∆C. The GFP-tagged protein is detected in the FITC channel (left), and telomeres are stained with an anti-TRF1 antibody (371, middle panels). The overlay is shown in the right panels.

In order to specifically screen for proteins that associate with the DNA binding domain of POT1, we constructed an allele predicted to contain the full OB fold necessary for DNA binding, but with a truncated TPP1 interacting domain. The locations of these domains have been extensively described and mapped [7,15,17]. The POT1 allele we constructed, termed POT1∆C, contains the first 379 amino acids of the protein, with a full DNA-binding domain as described in [7], with an additional 79 amino acids, but not the TPP1-binding domain, located in the C-terminal region [15]. Therefore, POT1∆C should be unable to quantitatively associate with telomeres but retain full DNA binding activity. Introduction of a MYC-tagged version of POT1∆C by retroviral transduction in HTC75 cells showed that this allele was expressed at significantly lower levels than full-length POT1 (1A, left). This was also observed in the context of a fusion with GFP, where GFP-POT1∆C showed lower levels than the GFP-POT1 wild-type fusion (Figure 1A, right). Despite low levels of expression, the GFP-tagged NLS-POT1∆C construct allowed us to assess the intranuclear localization of the protein. As predicted, and unlike full-length GFP-POT1, GFP-POT1∆C failed to accumulate to telomeres, but instead showed a diffuse nuclear pattern (Figure 1C). A GST-POT1∆C construct was made that allowed us to perform in vitro DNA binding assays. We found that the binding affinity of GST-POT1∆C was indistinguishable from that of the full-length protein (Figure1B). Therefore, the POT1∆C allele represents a segment of POT1 with a full DNA binding domain, suitable for expression in yeast as a two-hybrid bait. The screen was expected to yield clones that associate with the DNA binding domain of POT1, and to exclude TPP1, which interacts with the C-terminus of the molecule [15].

**Figure 2.**
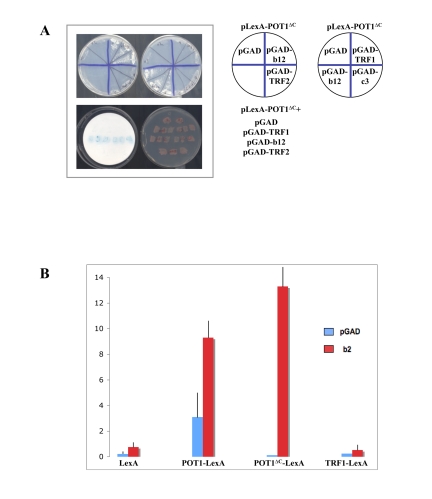
The LIM domains of TRIP6 interact with POT1∆C by yeast two-hybrid. (**A**) Top: His phenotypes of the B40 strain carrying the pLexA-POT1∆C bait plasmid and the plasmids shown on the right side, including the b12 positive clone and another recovered clone, c3, which proved negative upon retransformation. Bottom: Patch LacZ assay of the same strains, showing that the b12 clone activates the LacZ reporter gene as well. (**B**) Liquid β-Gal assays using the B40 yeast strain and the bait plasmids shown at the bottom, with the GAD vector or GAD-b2 clone. The b2 clone activates LacZ with LexA-POT1 or LexA-POT1∆C, but not with LexA-TRF1. The standard deviations were calculated on three independent yeast colonies, each assayed three times.

The yeast two-hybrid screen was performed with LexA-POT1∆C as a bait, in the L40 yeast strain, with the LacZ and HIS3 genes as reporters. After initial screening of 2x10^6^ transformants and subsequent retesting, 3 plasmids were recovered that conferred robust His+ and LacZ+ phenotypes upon re-transformation (Figure 2A). During the retests, plasmids containing TRF1-GAD, TRF2-GAD (Figure 2A, 2B) and RAP1-GAD (not shown), all fusions that were analyzed in separate studies in the L40 strain [26] [27], were also tested against the LexA-POT1∆C bait and showed no activation, demonstrating the specificity of the interaction for the bait. All three recovered clones corresponded to the 3' half of the same cDNA, containing the three C-terminal LIM domains of TRIP6 in fusion with the GAL4 activation domain at amino acid 218. Tests performed in yeast confirmed that the clones interacted with full-length POT1 (Figure 2B), showing that the interaction detected in yeast was not an artifact of the truncation of the protein. The activation of LacZ was evident with both POT1∆C and full length POT1, although in the latter case some background activation of the promoter occurred without the prey (Figure 2B). We also tested the library clone obtained against other LexA fusions such as LexA-TRF1 (Figure 2B), showing that the TRIP6 fusion had specificity for the POT1 bait. Altogether, the results from the two-hybp>rid screen argue for an interaction between the N-terminal domain of POT1 and the C-terminal LIM domains of TRIP6. A POT1∆OB fused to LexA construct was also tested against the TRIP6 fragment, in order to ask whether the interaction was lost when the first OB fold is absent. In this case, the high LacZ+ and His+ background caused by POT1∆OB alone precluded the analysis. TRIP6 was isolated previously as one of the thyroid receptor interacting molecules and was later characterized as binding to adhesion plaques. A role for organizing the actin cytoskeleton at adhesion plaques has been established [28], and nuclear roles for the protein have been described [29,30]. The molecule has a molecular weight of 60kD and can be divided into two roughly equivalent regions: an N-terminal half, containing a nuclear export sequence, referred to as the pre-LIM domain, and a C-terminal half with three predicted LIM domains. It is the latter region of TRIP6 which was isolated in the screen.

**Figure 3.**
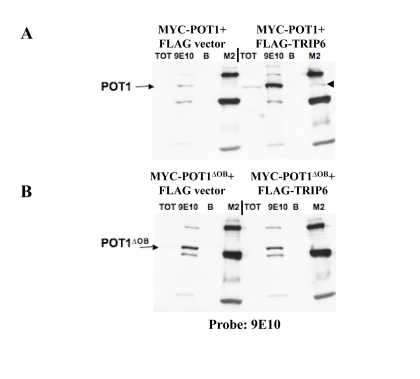
POT1 interacts with TRIP6 in transient transfections. (**A**) IP-Western blots on lysates made from transiently transfected 293T cells, to detect co-immunoprecipitation of the transfected proteins. MYC-POT1 was co¬transfected with FLAG-TRIP6 or with the FLAG vector, and the lysates were used for immunoprecipitations with the 9E10 (anti-MYC) or M2 (anti-FLAG) antibodies, as shown on top. A Total fraction (TOT) and beads only control (**B**) were run alongside. The blot was probed with the 9E10 antibody, and the position of MYC-POT1 is indicated by the arrow. The immunoprecipitated MYC¬POT1 by the FLAG antibody is shown with the black triangle. (**B**) Same as **A**, except that MYC-POT1∆OB is used in the co-transfection.

### Cloning and expression of the full-length human TRIP6 cDNA

The full-length TRIP6 cDNA was obtained as an EST and cloned into a retroviral mammalian expression vector (pLPC) in fusion with a FLAG or MYC epitope tag, in order to express the tagged full-length cDNA in human cells. We sought first to confirm the interaction between POT1 and TRIP6 detected in yeast. Transient co-transfections in 293T cells with full-length POT1, POT1∆C or POT1∆OB were performed to ask whether MYC-POT1 could coimmunoprecipitate specifically with FLAG-TRIP6. We found that TRIP6 could pull down full-length POT1 (Figure3A). In addition, co-transfection of TRIP6 resulted in the stabilization of POT1. Both these observations suggest an interaction between full-length TRIP6 and POT1. No interaction or stabilization was detected with POT1∆OB (Figure3B), suggesting, in conformity with the setup of the yeast two-hybrid screen, that the N-terminal OB folds are important for the POT1-TRIP6 interaction. It is possible, although not demonstrated here, that the first OB fold of POT1, which is missing in POT1∆OB, is necessary for the interaction. We also used MYC-POT1∆C in this assay. Owing to the low expression of the protein (see above), we could not detect an association with TRIP6 in this case. However, a stabilization of MYC-POT1∆C upon co-transfection with FLAG-TRIP6 was observed (not shown), compatible with an interaction between the two proteins.

**Figure 4.**
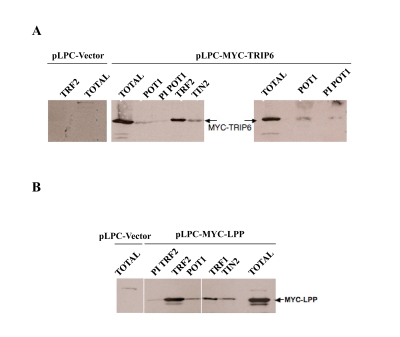
TRIP6 and LPP co-immunoprecipitate with several shelterin components. (**A**) IP-Western blots on lysates made from HTC75 cells obtained through retroviral transduction, stably expressing MYC-TRIP6 (50kD) (the vector only control is shown on the left). The lysates were used for immunoprecipitations with the antibodies listed on top, and analyzed for the amounts of MYC-TRIP6 by Western blot with the 9E10 antibody. The Total fraction was ran alongside as indicated. The POT1 sera were the anti-epitope #4955 (left panel), and the anti¬baculovirus POT1 #1048 (right panel). (**B**) Same as **A**, as with a MYC-LPP (66kD) expressing HTC75 cells.

A MYC-tagged TRIP6 cDNA was stably introduced by retroviral transduction into HTC75 cells, in order to further the co-immunoprecipitation analysis and to study the localization of the protein by immunofluorescence. In MYC-TRIP6 expressing cells, a weak but reproducible signal was detected after immunoprecipitation with two independent anti-POT1 sera (Figure 4A). This confirmed that the interaction detected by yeast two hybrid and transient transfection was detectable in stably expressing cells. We then explored whether TRIP6 could be pulled down with antibodies against other shelterin components. A very robust precipitation of TRIP6 was observed with an anti¬TRF2 antibody (Figure 4A). TRIP6 could also be pulled down with TIN2 antibodies (Figure 4A). Because TRIP6 could be pulled down by antibodies to several shelterin components, our results argue for an association between TRIP6 and the whole complex. We cannot rule out a direct interaction between TRIP6 and other shelterin components, such as TRF2, that are not detected by the yeast two-hybrid tests. The cellular localization of TRIP6 in our established cell lines was seen as mostly cytosolic staining, as previously described by others (SS and DL, unpublished, and [30]). We found no evidence for telomeric localization by immunofluorescence. We reasoned that accumulating TRIP6 in the nucleus might increase the signal detected in the co-immunoprecipitation experiments. To test this, we generated an allele of TRIP6 with an inactivated NES (the allele described in [30]), named TRIP6-NES . We confirmed by immunofluorescence that this mutant allele accumulated in the nucleus. No difference was observed in the coprecipitation between POT1 or TRF2 and wild-type or TRIP6-NES (not shown). It is possible that the interaction between TRIP6 and shelterin is highly regulated and not driven by high nuclear amounts of the protein.

### LPP, closely related to TRIP6, also interacts with shelterin

In our analysis of TRIP6, we noted that the human genome encodes a highly related protein called LPP, which, as TRIP6, is part of the Zyxin family. The homology in the C-terminal LIM domains between TRIP6 and LPP is very high: the sequence identity between the two in the first LIM domain is about 60%, and 77% and 75% for the second and third LIM domains respectively [31]. The high homology between the C-termini of TRIP6 and LPP prompted us to investigate whether LPP also associated with shelterin. Although the degree of identity is lower in the N-terminal third of the protein (around 35%), the overall domain structure is identical between LPP and TRIP6, with, notably, a nuclear export sequence arguing for active shuttling of LPP between cytoplasm and nucleus. Based on the degree of homology and domain structure of the two molecules, it is possible that TRIP6 and LPP share an ability to interact with shelterin. The full-length LPP cDNA was cloned into pLPC-MYC and stably expressing cell lines were obtained in HTC75. By IP-Western, MYC-LPP was found to co-precipitate with POT1, TRF2, TRF1 or TIN2 antibodies (Figure 4B).

Therefore, LPP could associate with shelterin as well. This shared ability with TRIP6 to be in a complex with shelterin could be mediated by the highly similar C-terminal LIM domains, although the interaction domains in TRIP6 and LPP remain to be defined.

### TRIP6 and LPP can be detected at telomeres by chromatin immunoprecipitation

Immunofluorescence analysis of TRIP6 or LPP localization yielded results in accordance with a previously published report [30]: the pattern displayed cytoplasmic staining compatible with a much higher concentration of TRIP6 and LPP in the cytoplasm than in the nucleus. We confirmed the published observation that TRIP6 or LPP could accumulate in the nucleus upon treatment with Leptomycin B supporting the notion that they both actively shuttle between nucleus and cytoplasm (SS and DL, unpublished).

**Figure 5.**
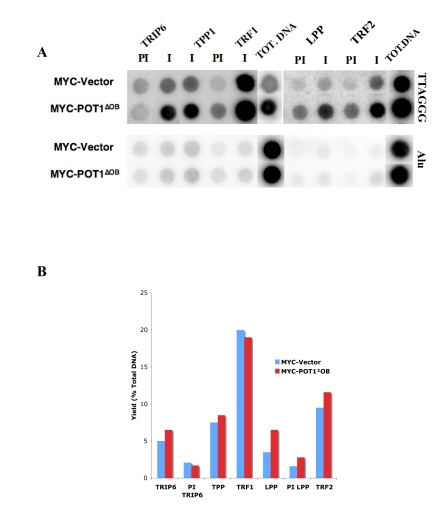
TRIP6 and LPP are detected at telomeres by ChIP. (**A**) Chromatin immunoprecipitations with fixed lysates prepared from HTC75 cell lines indicated on the left. The antibodies used are listed on top (I=Immune, PI=PreImmune), and the Total DNA fraction is on the right side of each blot as indicated. Extracted DNA samples were dot-blotted on Nitrocellulose, and probed with a TTAGGG probe (top), or with an Alu probe (bottom) as a control. The yields calculated for the samples probed with Alu were all below 0.5%. (**B**) Histogram of the values for the yields as % of total DNA of the samples shown in **A**.

In order to address whether TRIP6 or LPP associate with shelterin at telomeres, we turned to the chromatin immunoprecipitation (ChIP) technique. This technique has been extensively used to study the presence of shelterin components or other proteins on telomeric DNA. Anti-peptide rabbit sera against TRIP6 or LPP were used for this analysis, which were both raised against epitopes in the N-terminus which were significantly divergent between the two proteins. We confirmed that the TRIP6 and LPP sera were competent for immunoprecipitations and not crossreacting (Supplementary  Figure 2). In asynchronous HTC75 cells, TRIP6 was found to associate with telomeres with a yield of about 5% of total TTAGGG DNA (Figure 5A,5B), about half the yield seen for POT1 in this assay and comparable with the yield obtained for TPP1. TRF1 antibodies, used as a control here, pulled down 20% of total telomeric sequences, in accordance with previously published results [4]. LPP could also be detected at telomeres by ChIP (Figure 5A). The yield for LPP was 3.5% of total DNA, in the same range as TRIP6. The yields for Alu sequences, used her as internal control sequences, was between 0.5 and 1% for all samples. These results show that the interactions between TRIP6 or LPP and shelterin are taking place at the telomere and likely reflect a role for these LIM-domain proteins in telomere function. Thus, both TRIP6 and LPP are found at telomeres in asynchronously growing HTC75 cells.

We also probed the telomeric association of TRIP6 and LPP by ChIP in cells expressing POT1∆OB . These cells have highly elongated telomeres, concomitant with a lower expression of endogenous full-length POT1 [4]. We found that TRIP6 or LPP show a yield similar to that observed in non-expressing HTC75 cells (ca 5%) (Figure 5A, 5B), which results in a stronger signal on the dot-blot (Figure 5A) due to the significantly longer telomeres in POT1∆OB cells. Such a pattern is observed for other telomeric or telomere-associated proteins such as TRF1 (see Figure 5A), POT1, RAP1 or MRE11, and is evidence for association with the overall telomeric chromatin [4]. Therefore, it appears that TRIP6 and LPP associate with shelterin along the whole telomere. Also, the strong depletion of full length, endogenous POT1 in POT1∆OB cells [4] does not lead to a disappearance of TRIP6 or LPP from telomeres, suggesting that the OB folds of POT1 are not involved in recruiting TRIP6 or LPP to telomeres, which would then occur through other events or interactions to be defined. Thus, the interaction between the LIM domains of TRIP6 and the N-terminus of POT1 is not expected to mediate the recruitment of TRIP6, but rather to be relevant to the function of the protein. Whether the same holds true for LPP remains to be determined, but the high homology between the LIM domains of LPP and TRIP6 suggest that they both are able to interact with the POT1 N-terminus. The modalities of recruitment of TRIP6 and LPP to telomeres are interesting questions to pursue.

### TRIP6 and LPP are involved in telomere protection

To analyze the possible roles of TRIP6 and LPP in telomere function we first examined telomere length in HTC75 cells over 60 population doublings in cells overexpressing either TRIP6 or LPP. The impact of shelterin components depletion or overexpression is normally detected during this span, but no effect was observed for TRIP6 or LPP (data not shown). We then turned to the analysis of TRIP6 or LPP siRNA depletion on telomere protection in HTC75 cells.

**Figure 6.**
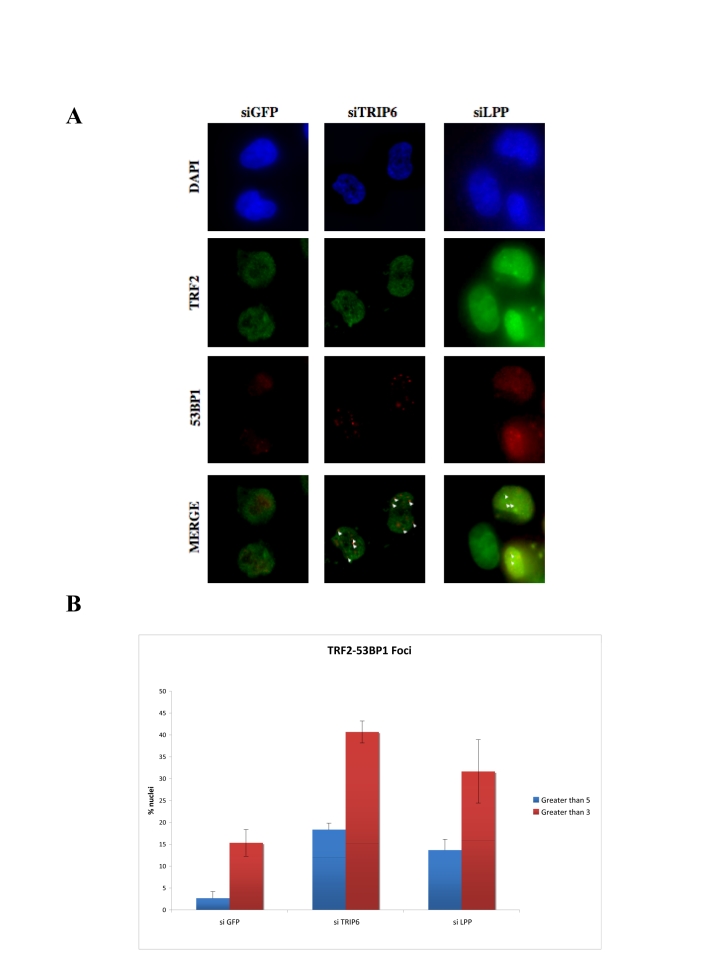
Depletion of TRIP6 or LPP leads to TIF formation. (**A**) Immunofluorescence showing the intranuclear localization of p53BP1 in TRIP6¬depleted HTC75 cells (middle panels), LPP-depleted cells (right panels) or control siRNA (GFP, left). The detected fluorescence (DAPI, FITC for TRF2, TRITC for p53BP1) is indicated on the left, and white triangle point to the co¬localized TRF2-p53BP1 foci. (**B**) Histogram of the values for co-localized p53BP1 and TRF2 foci (left, greater than 3, right, greater than 5 per nucleus) as a percent of the total nuclei counted. 100 nuclei were counted for each set, and the standard deviations were calculated on three separate experiments.

We analyzed the possible short-term effects (48hr post transfection) of TRIP6 or LPP depletion on the induction of a DNA damage response at telomere. Such a response can be monitored by the induction of p53BP1 foci that partially co-localize with telomeres, indicative of telomere de-protection [18,32]. For siRNA of TRIP6 or LPP, we used targets sites that led to partial depletion of exogenous MYC-TRIP6 or MYC¬LPP as observed by Western blot (Supplementary Figure 1). The depletion of TRIP6 led to a significant increase of p53BP1 foci in the nuclei, suggestive of an induction of a DNA damage response a numerous sites in the genome. The number of p53BP1 nuclear foci increased from an average of 1 to 2.65 per nucleus, including untransfected cells which tend to lower the number in this case. In particular, we observed the induction of telomere dysfunction induced foci (TIFs), as observed by the formation of p53BP1 foci that co-localized with TRF2 (Figure 6). Upon depletion of TRIP6, 40% of the nuclei showed 3 or more p53BP1 foci co-localizing with TRF2, indicating that some of these foci represented a DNA damage response at telomeres. This value represented a 2.7-fold increase over background, detected in the GFP siRNA control.

We observed similar results with the siRNA depletion of LPP. In this case also, an overall increase of p53BP1 was evident, from 1 to 1.8 foci per nucleus, arguing for a role in general repression of a DNA damage response in these cells. The degree of TIF formation was similar, but slightly lower, to that observed with TRIP6, with 32% of the cells showing 3 or more foci co-localizing with TRF2, a two-fold increase over background. Since depletion of either TRIP6 or LPP alone led to a DNA damage response at telomeres, we conclude that both are necessary to fully protect telomeres, possibly by cooperating with POT1.

## Discussion

In this study, we describe a novel association between shelterin and LIM domain proteins at telomeres. These are the LIM-domain proteins TRIP6 and LPP, two related molecules of the Zyxin family [25]. We first identified TRIP6 in a two-hybrid screen for proteins that associate with the DNA binding domain of POT1. Binding to POT1 and the shelterin complex could have two significant consequences: the recruitment of the protein to telomeres, and a role in telomere function. Given that the DNA binding domain of POT1 mediates the function of the protein in protection and length regulation [1], and that TRIP6 and LPP recruitment are not affected by high expression of POT1∆OB, we argue that the interaction between POT1 and TRIP6 detected in yeast relates to function and not recruitment. It remains to be determined how and when TRIP6 and LPP are recruited to telomeres.

The LIM superfamily of proteins contains at least 50 members in the human proteome and is subdivided into seven families, all made of proteins with predicted LIM domains in various arrangements [25]. TRIP6 is part of the Zyxin family, along with other members such as LPP or Ajuba, characterized by the presence of three LIM domains at the C-terminus of the molecule. However, intriguingly, these molecules possess a nuclear export sequence, which accounts for their active shuttling between the nucleus and the cytoplasm. A nuclear role for TRIP6 as a transcription factor has been described [29,30], arguing for an important role for this molecule in addition to that performed in the cytoplasm.

TRIP6 and LPP are not detected at telomeres by immunofluorescence, and, instead, show a cytoplasmic localization pattern seemingly at odds with a role in the nucleus and at telomeres (DL and SS, unpublished, and [30]). However, they are known to shuttle actively between the cytoplasm and the nucleus, in a manner dependent on the NES present in the N-terminal half of the molecule [30]. The telomeric association we detect is therefore probably not representative of the majority of the cells in the population, but rather occurs in a minority of the cells experiencing high TRIP6/LPP nuclear concentration. Although this remains to be established, it would be interesting to investigate an accumulation of TRIP6 and LPP at telomeres during S-phase, a period in the cell cycle with high demand for protective activities [33]. As such, TRIP6 and LPP would be active only transiently at telomeres, perhaps during DNA replication.

The roles of TRIP6 and LPP could impact on two main processes: either telomere protection or telomere length regulation through the control of telomerase. Our results suggest that TRIP6 and LPP both individually contribute to the protection of telomeres, in preventing the damage response otherwise elicited through activation of ATM or ATR pathways. An impact for TRIP6 or LPP on telomere length regulation has not been detected but is still under investigation. The high sequence similarity between TRIP6 and LPP likely accounts for their recruitment to telomeres, possibly through a common pathway, although our data argue that each individually is important for proper telomere protection.

A nuclear role for LIM proteins, in particular in the Zyxin family, has been established previously. Like TRIP6, the protein Ajuba, has an NES as well as three C-terminal LIM domains. Ajuba was found to associate with kinetochores and to participate in the spindle assembly checkpoint [34]. Also, Ajuba was found to co-repress transcription at RAREs through interactions with, among other factors, RARα. The mechanism of co¬repression was found to occur through recruitment of PMRT5, an Arginine methylase whose enzymatic activity was found to be essential in this process [35]. In this context, the LIM domains of Ajuba constitute a platform of interactions to mediate transcriptional repression through Arginine methylation. Interestingly, Ajuba shares the ability to interact with RARα with two other Zyxin family members, WTIP and LimD1, while TRIP6 and LPP are negative in this assay [35]. This observation parallels ours, in that more than one LIM protein can play a role in the same process. We find it tempting to speculate that TRIP6 and LPP bring similar activities to the telomeres, as Ajuba and WTIP do to RAREs, in recruiting for instance an Arginine methylase. Arginine methylation of TRF2, a shelterin component, has recently been implicated in telomere function, in particular in repressing premature sense-cence in primary cells [36]. Therefore, there might be a subdivision in the Zyxin family, some members being involved in transcriptional repression, and others in telomere protection. Further work will determine whether the activities of TRIP6 and LPP at telomeres are important for the repression of the senescence path-way under the control of shelterin, in particular TRF2.

## Materials and methods

Two-hybrid screen/Isolation of TRIP6. A two-hybrid screen was carried out with the yeast reporter strain L40 (Hollenberg et al., 1995; Bianchi et al., 1997), using the human POT1∆C C-terminal truncation (aa 1-379) fused to the LexA DNA binding domain as a bait. The L40 strain bears the HIS3 and LacZ reporter genes under the control of the LexA DNA binding site. The libraries used were the HeLa S3 or the human testis matchmaker cDNA library (Clontech), containing random fusions to the GAL4 activation domain. The TRIP6 two-hybrid cDNA clones (aa positions) containing the three LIM domains of the molecule where isolated and re-transformed into the L40 containing the bait to ensure that the His+ and LacZ+ phenotypes were due to the library plasmid.

Two-hybrid assays. The two-hybrid tests shown in Figure 2 were performed also in the L40 strain with the LexA-TRIP6 full length cloned in pBTM116 (Bartel et al., 1993) by PCR of the TRIP6 EST (see below) and tested against a number of previously characterized and published fusions with the GAL4 activation domain: TRF1-GAD, TRF2-GAD, and POT1¬GAD, all cloned into the pACT2 vector (Clontech). The BGal liquid assays were performed as described in the Clontech Matchmaker protocol and three independent colonies were assayed for each plasmid combination, and the standard deviations reported are based on three separate experiments.

Cell lines and antibodies. The HTC75 cell line is a HT1080 derivative described in [37]. The cells were grown in DMEM/10%BCS, and the retroviral transduction protocol was identical to that described in [38]. The antibodies against TRIP6 and LPP were generated against a peptide conjugated to KLH and used for immunization into rabbits, as per the protocol set by the manufacturer (BioSynthesis, Lewisville, TX). The peptides were: NH2-GCPKKFAPVVAPKPKYNPYKQ -OH for LPP, and GC-LNGGRGHASRRPDRQAYE-OH for TRIP6. The TRF2 antibodies were the 647 against the full-length protein made in Sf9 cells [11], or the anti-peptide 508 [39]; the TRF1 antibody was the anti-peptide 371 [37]; the POT1 antibodies were the 1048 against the full-length protein made in Sf9 cells or the anti-peptide 978 [4]; and the Tin2 antibodies were the 864 made against the full-length protein in Sf9 cells [40]. The p53BP1 antibody was purchased from Novus (NB100-304).

Plasmids. The TRIP6 and LPP cDNAs were purchased as full length clones from the EST collection maintained by the ATCC (TRIP6) or Invitrogen (LPP). The full length cDNAs were amplified by PCR using primers with appropriate cloning sites (5' Bgl II and 3' Xho I) and cloned into pLPC-MYC [38] to generate a MYC tagged version driven by the CMV promoter. The PCR oligonucleotides were: 5' AGATCTTCGGGGCCCACC TGGCTGCCCCCG and 5'CTCGAGTCAGCAGTCAG TGGTGACGGTGGC for TRIP6, and 5' AGATCTCA CCCATCTTGGC and 5' GAGTCTGAGCTAAAGGT CAGT for LPP.

The POT1∆OB construct is described in [4], and the POT1∆C construct was cloned by PCR-cloning of amino acids 1-379 of POT1 into pLPC-MYC for expression into human cells, or pBTM116 to use as a bait for the two-hybrid screen. The POT1 and POT1∆C fusion with eGFP where performed using a vector constructed with the eGFP fragment from the pEGFP-C1 vector (Clontech) subcloned into pBabe-Puro. An NLS was cloned as a BamHI fragment into the BglII site of the polylinker, and the full-length POT1 or POT1∆C fragments were cloned as BamHI-XhoI fragments downstream of the NLS, generating N-terminally GFP-tagged protein fusions actively transported into the nucleus.

RNA interference. HTC75 cells were maintained in DMEM (Invitrogen) supplemented with 1% penicillin and streptomycin and 10% fetal bovine serum (FBS). TRIP6, and LPP-speciﬁc siRNAs were synthesized by Dharmacon RNA Technologies. For TRIP6 RNAi, double-stranded siRNA were designed to target the following sequences: TRIP6(6.1)siRNA 5'-AGGAGGA GACUGUGAGAAUUU-3' TRIP6(6.2)siRNA 5'-CUGGAUAGGCUGACGAAGAUU-3' LPPsiRNA(L.1) 5'CUCAUAAUGUGAAAUAUGA¬3' LPPsiRNA(L.2) 5'GCCAUUCUAUGCUGUGGAA-3' HTC75 cells were transfected using Lipofectamine (Invitrogen) according to the manufacturer's instructions. Briefly, cells at a confluency of approximately 50-60% were plated in a 6-well plate 18-24 hr prior to transfection. Transfections were done one time within a 24 hr interval and cells were processed 48 hr after the first transfection. As a control, siRNA designed to target GFP (Dharmacon) was used.

Immunofluorescence. Immunostaining for TRF2 and 53BP1 proteins was performed on HTC75-Vector, MYC-TRIP6 or MYC-LPP cells plated onto glass coverslips. Cells were fixed with 2% formaldehyde in PBS (v/v) for 10 min at RT. Cells were permeabilized with 0.5% NP40 in PBS for 10 min at RT, washed two times in 1X PBS, and blocked with PBG for 30 minutes. Coverslips were then incubated with the mouse anti-TRF2 antibody clone 4A794 (Millipore/Upstate Biotech) and a rabbit anti-p53BP1 antibody (Novus NB100-304A-1), both at a concentration of 1:1000 in PBG overnight.

Cover slips were then rinsed three times with PBG solution and incubated with secondary TRITC-conjugated goat anti-rabbit antibody or FITC-conjugated donkey anti-mouse antibody (Jackson Immunoresearch) in PBG at a concentration of 1:1000 for 45 min at RT. Cover slips were rinsed two times with PBG. Coverslips were then incubated with PBG and 4=,6=-diamidino-2-phenylindole (DAPI) at 100 ng/ml to visualize the nuclei. Coverslips were mounted on to slides with embedding media. Images were collected with an Olympus BX61 fluorescence microscope using a 60X objective connected to a Hamamatsu ORCA-ER CCD camera, controlled by the SlideBook 5.1 image capture software.

Chromatin immunoprecipitations . The chromatin im-munoprecipitations were performed as described in [4].

E.coli GST-POT1 and bandshift assays . The purifica-tion protocol is detailed in [41], and the bandshift assays were performed as described in [6].

## Supplementary data

Supplementary Figure 1 Depletion of TRIP6 or LPP by siRNA. Western blot of lysates prepared from MYC-TRIP6 or MYC-LPP expressing cells transfected with the siRNA indicated on top, with the anti-MYC 9E10 antibody as a probe.  The siRNA 6.2 was used for TRIP6 depletion, and P.1 for LPP depletion.

Supplementary Figure 2 The anti-TRIP6 or LPP sera are proficient for immunoprecipitations. IP-Western blots showing that the rabbit TRIP6 antibodies (5023) or LPP antibodies (6073,6074) are able to immunoprecipitate MYC-TRIP6 or MYC-LPP, with the preimmune sera (PI) as negative controls. The TRIP6 sera did not precipitate LPP, and the LPP sera did not precipitate TRIP6.
