# Supplementary figures and images for: LIM-domain proteins TRIP6 and LPP associate with shelterin to mediate telomere protection
Source: Aging (Albany NY). 2010 Jul 14;2(7):432–44. doi: 10.18632/aging.100170 (PMC2933890; doi:10.18632/aging.100170)

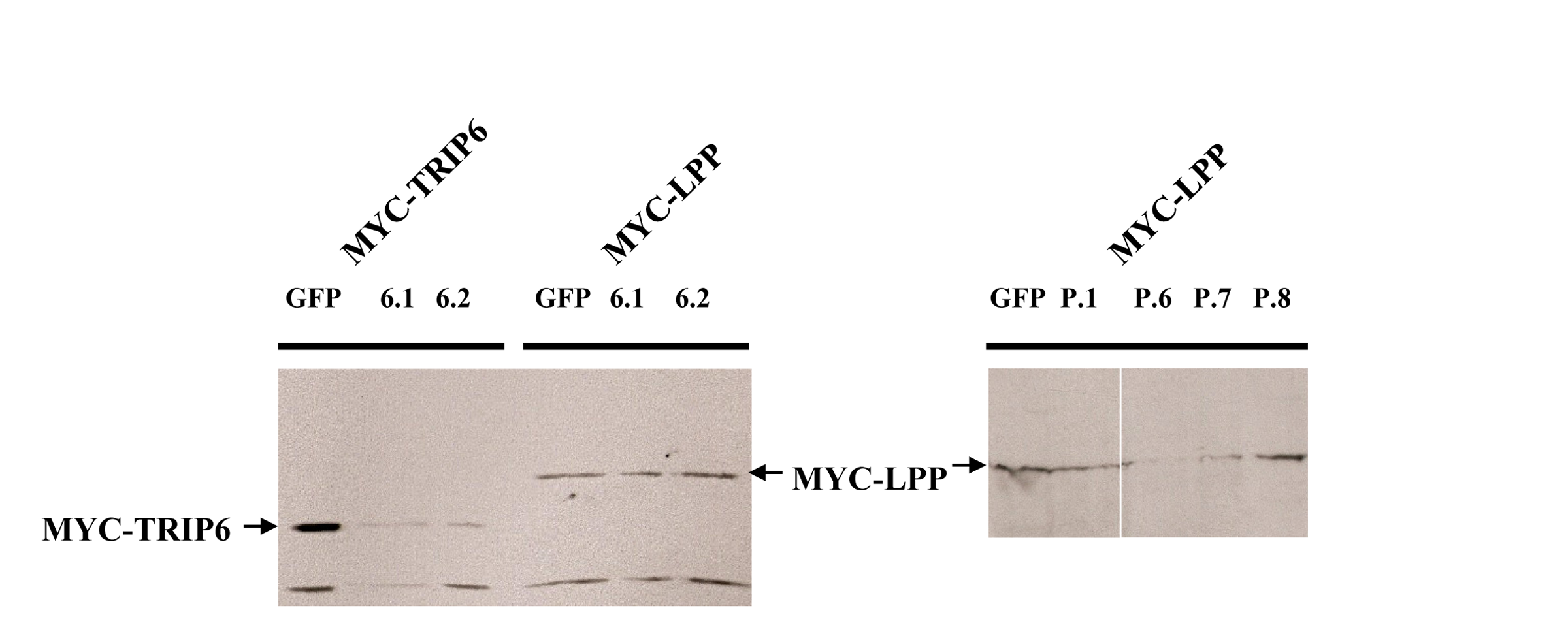

Supplement: Supplementary Figure 1 — Western blot of lysates prepared from MYC-TRIP6 or MYC-LPP expressing cells transfected with the siRNA indicated on top, with the anti-MYC 9E10 antibody as a probe. The siRNA 6.2 was used for TRIP6 depletion, and P.1 for LPP depletion. [file aging-02-432-s001.tif]

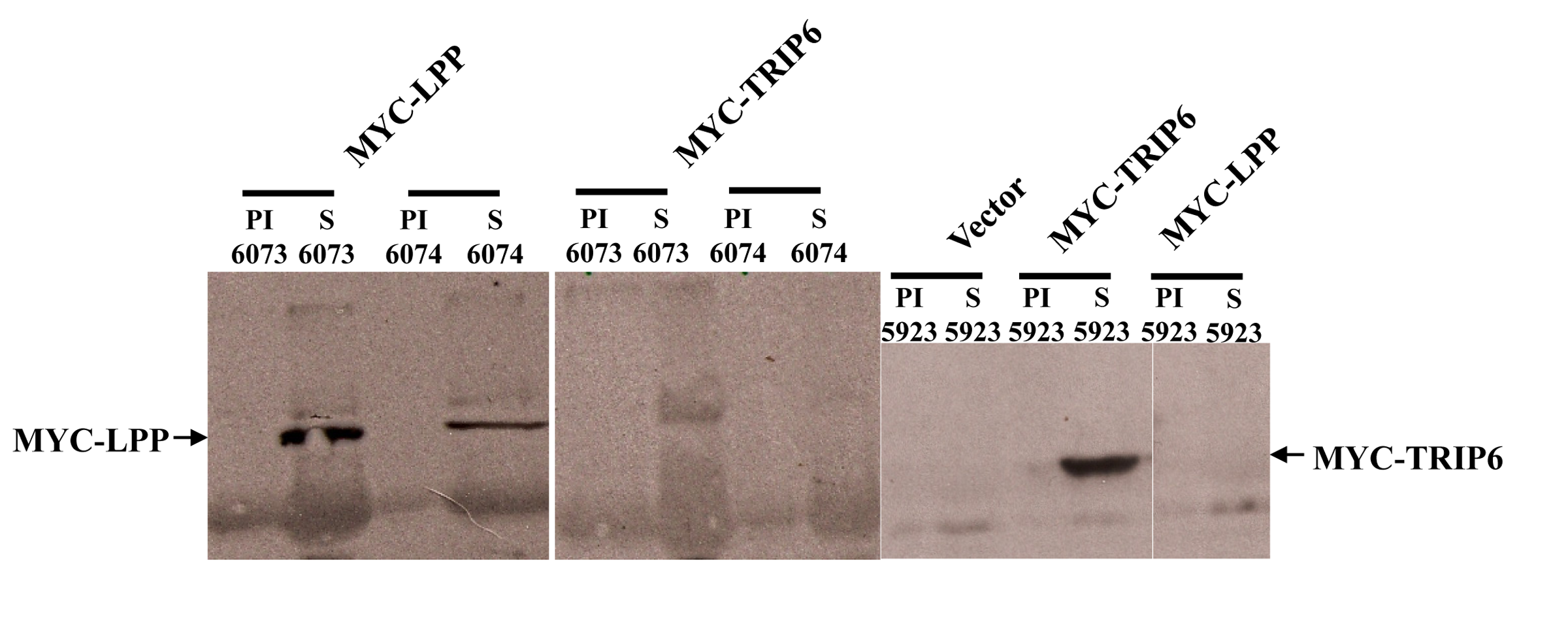

Supplement: Supplementary Figure 2 — IP-Western blots showing that the rabbit TRIP6 antibodies (5023) or LPP antibodies (6073,6074) are able to immunoprecipitate MYC-TRIP6 or MYC-LPP, with the preimmune sera (PI) as negative controls. The TRIP6 sera did not precipitate LPP, and the LPP sera did not precipitate TRIP6. [file aging-02-432-s002.tif]
